# A point mutation in HIV-1 integrase redirects proviral integration into centromeric repeats

**DOI:** 10.1038/s41467-022-29097-8

**Published:** 2022-03-18

**Authors:** Shelby Winans, Hyun Jae Yu, Kenia de los Santos, Gary Z. Wang, Vineet N. KewalRamani, Stephen P. Goff

**Affiliations:** 1 Department of Biochemistry and Molecular Biophysics, Columbia University Medical Center, New York, NY USA; 2 Department of Microbiology and Immunology, Columbia University Medical Center, New York, NY USA; 3 Howard Hughes Medical Institute, Columbia University, New York, NY USA; 4 Basic Science Program, Leidos Biomedical Research, Frederick National Laboratory, Frederick, MD USA; 5 Department of Pathology, Columbia University Medical Center, New York, NY USA; 6 Basic Research Laboratory, Center for Cancer Research, National Cancer Institute, Frederick, MD USA

**Keywords:** DNA, Retrovirus, DNA recombination, Transposition

## Abstract

Retroviruses utilize the viral integrase (IN) protein to integrate a DNA copy of their genome into host chromosomal DNA. HIV-1 integration sites are highly biased towards actively transcribed genes, likely mediated by binding of the IN protein to specific host factors, particularly LEDGF, located at these gene regions. We here report a substantial redirection of integration site distribution induced by a single point mutation in HIV-1 IN. Viruses carrying the K258R IN mutation exhibit a high frequency of integrations into centromeric alpha satellite repeat sequences, as assessed by deep sequencing, a more than 10-fold increase over wild-type. Quantitative PCR and in situ immunofluorescence assays confirm this bias of the K258R mutant virus for integration into centromeric DNA. Immunoprecipitation studies identify host factors binding to IN that may account for the observed bias for integration into centromeres. Centromeric integration events are known to be enriched in the latent reservoir of infected memory T cells, as well as in elite controllers who limit viral replication without intervention. The K258R point mutation in HIV-1 IN is also present in databases of latent proviruses found in patients, and may reflect an unappreciated aspect of the establishment of viral latency.

## Abstract (web summary)

HIV-1 integration sites are biased towards actively transcribed genes, likely mediated by binding of the viral integrase (IN) protein to host factors. Here, Winans et al. show that the K258R point mutation in IN eredirects viral DNA integration to the centromeres of host chromosomes, which may affect HIV latency.

## Introduction

Insertion of retroviral DNA genomes into the host cell genome, a process termed integration, is an obligate step of a successful retroviral infection. By permanently integrating the viral genome into the host genome, retroviruses are able to persist indefinitely in the infected cell as a provirus. Integration is catalyzed by the virally encoded integrase (IN) protein^1,2^. Although all of the host genome is available as a target for integration at some frequency, the distribution of integration sites across the genome is not completely random and various retroviruses exhibit distinct integration site preferences^3^. These differential integration site selectivities can be primarily explained by the binding of the viral IN protein to various host factors^4^. Human immunodeficiency virus (HIV-1) has a significant preference for integrating into active gene regions^5^, and this preference is in part due to binding of the IN protein to the host factor LEDGF, a general transcriptional activator^6–9^. Similarly, the BET family of proteins has been shown to bind to murine leukemia virus (MLV) IN and target integrations near transcription start sites^10,11^. These host factors are believed to act largely as bimodal tethers, binding both the viral IN protein and host chromatin, and thereby biasing integration sites to specific genomic regions^12–14^.

While interactions of IN with host factors are important in targeting integration, recent work has also demonstrated that the retroviral structural protein, capsid (CA), is retained in the HIV-1 pre-integration complex (PIC) and also plays a role in integration site selection^15–17^. In HIV-1 infection, CA was shown to bind the host factor CPSF6. Blocking this association by mutation led to a significant redirection of integration sites, suggesting a potential role for CA in influencing integration targeting toward gene-dense regions of the genome^18^. For other retroviruses, such as MLV and the spumaretrovirus prototype foamy virus (PFV), structural proteins derived from Gag appear to directly bind chromatin and affect the overall efficiency of integration^19,20^. The MLV p12 protein, a cleavage product of Gag precursor, has been shown to tether the MLV PIC to mitotic chromosomes through direct binding to host nucleosomes^20^. Mutation of p12 to ablate chromatin binding results in a dramatic 100-fold decrease in detectable integrated proviruses, which can be partially rescued by introduction of a heterologous chromatin binding protein^20^. This suggests that the association functions as a mechanism to tether the PIC to chromosomes during mitosis when the nuclear envelope is broken down and thereby bypass a need for active nuclear import. Similarly, the Gag protein of PFV binds directly to host histone proteins and tethers the PIC to mitotic chromosomes^19^. Mutation of a single residue of PFV Gag known to interact with histones results in a global redistribution of integration sites towards centromeres, suggesting a role for Gag in the process of integration site selection^19^.

Integration targeting by chromatin tethering is a conserved mechanism amongst retroviruses and retrotransposons alike. The yeast Ty elements in particular exhibit highly specific integration targeting, down to the nucleotide in some cases^21^. Ty5 elements are mainly integrated into heterochromatic regions at telomeres or the mating type loci through interaction of the Ty5 IN protein with the yeast silencing factor Sir4^22,23^. The affinity of Ty5 IN for Sir4 is dependent on phosphorylation of the targeting domain of IN^24^. In the absence of IN phosphorylation, as occurs during certain stress conditions, Sir4 binding is lost and Ty5 integration is dramatically redirected in a dispersed fashion throughout the yeast genome^24^.

In this work, we demonstrate a dramatic redirection of integration site distribution induced by a single point mutation in HIV-1 IN. Viruses carrying the K258R mutation in the IN protein integrate into centromeric alpha satellite repeat sequences at more than ten times the frequency observed for WT virus, as assessed by next generation sequencing (NGS) of integration sites. The integration bias of the K258R mutant virus was further confirmed using quantitative PCR and in situ immunofluorescence assays. A mass spectrometry study following immunoprecipitation identifies host factors binding to IN that may account for the observed bias for integration into centromeres. Centromeric integrations of HIV-1 have been observed in latent infection as well as in elite controllers. The K258R mutation has also been observed in databases of latent proviruses suggesting an unappreciated role in establishing viral latency.

## Results and discussion

**Fig. 1:**
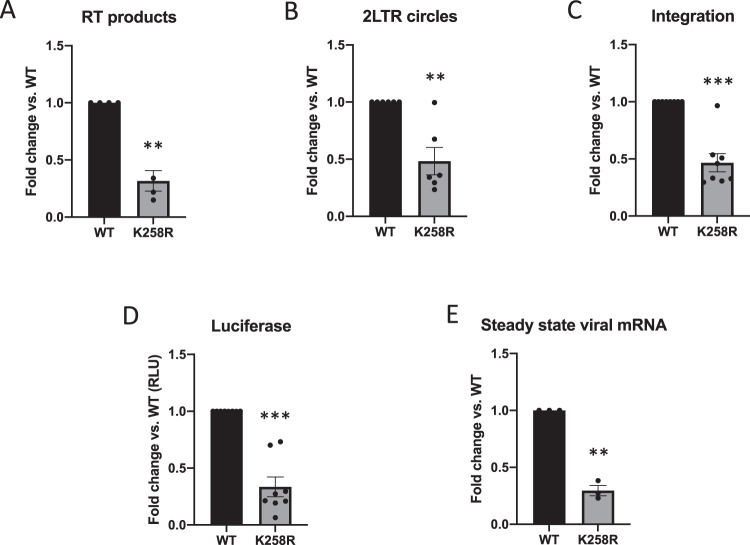
K258R point mutation in HIV-1 IN has modest effects on early viral replication. HeLa cells were infected with virus generated from pNL4.3.Luc.R-E- carrying either WT or K258R mutant IN. Infected cells were collected at 2 days post-infection. Abundance of (**A**) reverse transcription (RT) products and (**B**) 2-LTR circles was determined by qPCR and normalized to a housekeeping gene (*n* = 4 and 6 respectively). **C** Proviral integration frequency was assayed using a nested PCR Alu-gag approach (*n* = 7). **D** Luciferase activity was measured (RLU) and normalized by protein content to adjust for number of cells in input sample (*n* = 8). **E** Steady state viral mRNA levels were measured by qPCR of infected cellular cDNA using primers against spliced *tat* message (*n* = 3). All data is shown as a fold change relative to WT and is the average of the indicated number of independent biological replicates ± SEs. Statistical significance was gauged by two-tailed paired *t* test (*p < 0.05; **p < 0.01; ***p < 0.001). Source data are provided as a Source Data file.

HIV-1 IN is known to be heavily post-translationally modified, but no evidence to date has linked any post-translational modifications to integration site selection^25,26^. There are four major acetylation sites in the C-terminal domain (CTD) of HIV-1 IN (K258, K264, K266, and K273)^27,28^. We mutated these lysine residues to charge-conservative arginines, either singly or in combination. We generated pseudotyped single-round infection HIV-1 viral reporter constructs expressing luciferase, packaged into virion particles with either a WT IN or a mutant IN, and used them to transduce cells in culture. Infected cells were collected at 48 h post-infection and assayed for successful viral transduction by quantifying viral DNA products as well as luciferase activity (Fig. 1). We have previously examined the effects of these mutations on viral transduction, and reported that mutation of all acetylated lysine residues in combination led to a dramatic decrease in proviral transcription immediately after viral DNA integration^29^. In this study, we focus specifically on the K258R point mutation in HIV-1 IN.

The K258R point mutation in IN caused only a very modest threefold defect in total reverse transcription (RT) as gauged by qPCR quantification of viral DNAs (Fig. 1A), and an equally modest twofold decrease in the abundance of 2-LTR circular DNA, a structure generated upon nuclear entry (Fig. 1B). The mutation resulted in a similar twofold reduction in the levels of proviral DNA formed after infection as compared to WT, measured by qPCR amplification of host-viral junctions (so-called Alu-gag assays; Fig. 1C). Quantification of luciferase activity and steady state viral mRNA transcripts corroborated a similar decrease in overall viral transduction (Fig. 1D, E). These findings indicate that all viral DNA intermediates and viral mRNA levels are reduced by a comparable amount in the cells infected with virus carrying the K258R IN mutation, and that there is no significant defect at the specific step of integration. The small decrease in transduction is accounted for by the initial decrease in RT products and thus in viral DNA available for subsequent steps.

Based on the alteration of integration site distribution induced by changes in phosphorylation status of the retrotransposon Ty5 IN in yeast, we mapped integration sites produced by the acetylation mutant INs as compared to WT IN. We used PCR and high-throughput DNA sequencing methods to recover and characterize viral–host genome junctions. Integrations were then mapped to unique human sequences using Bowtie2 and analyzed for correlation with RefSeq genes, CpG islands, transcription start sites, DNase hypersensitivity sites and various protein or histone binding sites identified in ChIP-seq data sets. These alignments are restricted to single- or low-copy number genomic sequence databases.

**Fig. 2:**
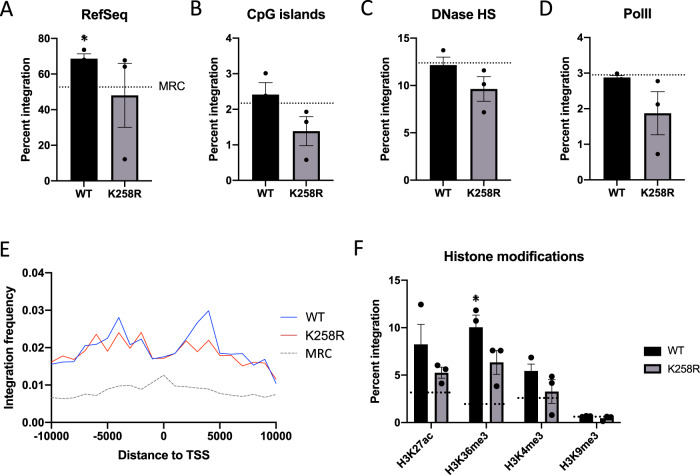
The K258R mutation in IN has minimal effect on integration site distribution into low-copy number sequences. Integration sites were mapped to the GRCh38 human reference genome assembly using Bowtie end-to-end alignment. Frequency of integrations falling within (**A**) RefSeq genes, or within 1 kb of (**B**) CpG islands, (**C**) DNase hypersensitivity sites, and (**D**) RNA polymerase II binding sites was calculated using BedTools. The frequency of integrations expected to be located near these features by random chance (matched random control, MRC) is shown as a dashed line. **E** Distribution of integrations around transcription start sites (TSS). Integrations in a 10 kb window around TSS are shown. **F** Frequency of integrations within 1 kb of select pre-infection histone modification sites. Data shown is the average of three independent biological replicates ± SEs. Statistical significance of average integration frequency relative to MRC as gauged by a one-sample, two-tailed *t* test is shown (*p < 0.05). Additional statistical analysis comparing the integration site pattern of WT and K258R mutant IN is shown in Table S1. Source data are provided as a Source Data file.

The combinatorial quadruple acetylation (QA) mutant IN and three of the four point mutant INs produced proviral integration patterns with very little deviation from the WT pattern (Fig. S1). However, we observed significant differences in the distribution of proviruses integrated at uniquely mapped sequences by the K258R mutant IN as compared to those formed by WT IN regardless of whether the NGS data was assessed in cumulative fashion (Table 1) or as the averages of three independent experiments (Fig. 2). As previously shown, WT HIV-1 proviruses were preferentially located in annotated RefSeq genes^5^. The K258R mutation reduced this preference for integration into genes to below the level of random chance (matched random control, MRC) (Fig. 2A). Similarly, the WT IN showed the expected slight preference for integrating near CpG islands, but the K258R mutant IN showed less of this preferential targeting (Fig. 2B). This general reduction in integration frequency near these sites held true for other genomic features as well, including DNase hypersensitivity regions and RNA polymerase II binding sites (Fig. 2C, D). These decreases were not due to an overall decrease in integration frequency, since all quantifications were normalized to the total number of unique integrations mapped. The distribution of integration sites relative to transcription start sites, however, was unchanged by the K258R mutation (Fig. 2E). We also correlated proviral integration sites to the genomic coordinates of various pre-infection histone modifications present in HeLa cells (Fig. 2F). We observed no notable differences in the frequency of proviral integration sites occurring in proximity to any of four pre-infection chromatin modifications (H3K27ac, H3K36me3, H3K4me3 and H3K9me3) generated by the K258R mutant IN as compared to WT (Fig. 2F). We did not observe a strong consensus sequence at the site of integration by WT IN, consistent with earlier reports, and the pattern was not substantially different at integration sites produced by the K258R mutant (Fig. S2).

**Table 1:** Sum of unique integrations mapped in three biological replicates.

	WT	K258R	Random
Unique integrations	17,636	18,850	10,000
Within RefSeq genes	12,363 (70.1%)	11,737 (62.3%)	5271 (52.7%)
TSS (5 kb)	3630 (20.6%)	3412 (18.1%)	2316 (23.2%)
TSS (1 kb)	671 (3.8%)	387 (2.1%)	668 (6.7%)
CpG islands (5 kb)	2549 (14.5%)	2291 (12.2%)	760 (7.6%)
CpG islands (1 kb)	426 (2.4%)	316 (1.7%)	217 (2.2%)
RNA Pol II (1 kb)	501 (2.8%)	447 (2.4%)	295 (2.95%)
DNase HS (1 kb)	2048 (11.6%)	2018 (10.7%)	1238 (12.4%)
H3K27ac (1 kb)	1197 (6.8%)	1080 (5.7%)	527 (5.3%)
H3K36me3 (1 kb)	1656 (9.4%)	1379 (7.3%)	326 (3.3%)
H3K4me3 (1 kb)	875 (5.0%)	809 (4.3%)	432 (4.3%)
H3K9me3 (1 kb)	121 (0.7%)	108 (0.6%)	102 (1%)
Within centromeres	146 (0.8%)	1576 (8.4%)	160 (1.6%)

The cumulative percent of integration seen within each feature is indicated.

The analysis of the distribution of integrations of mutant K258R IN into unique mappable sites showed a loss of selective targeting to active genes as well as other features, but did not reveal a concomitant increase in integration frequency elsewhere. To examine the distribution of integration sites more globally and determine where the K258R mutant IN is being redirected, we made use of scan statistics to identify regions of the genome with high numbers of viral integrations in an unbiased fashion, and specifically including highly repetitive sequences^30^. We analyzed common sites of integration or “hot spots” using a custom perl script^31,32^. This script first removes identical reads resulting from potential PCR duplication. Reads with identical viral–host genome junction sequences but disparate read lengths (breakpoints) were condensed into a single event. To account for potential copying errors induced by multiple rounds of PCR or sequencing we also combined those integrations in which the host sequence had >95% similarity over the length of the read. We then used a sliding window to scan the human genome for common sites of integration. For our purposes hot spots were defined as 5 or more integrations in a 10 kb window.

We identified an unprecedented number of hot-spot sites for integration by the K258R mutant IN that all clustered in centromeres (Table 2). The frequency of insertion of the mutant into centromeric regions was extraordinarily high, with 10 clear genomic hot spots of integration. WT HIV-1 IN has been previously reported to disfavor integration into centromeric repeats^5,33^, and indeed there were no such detectable integration hot spots in cells infected with WT HIV-1 virus. The clustering we observe in the K258R mutant integration distribution could not be attributed to selective outgrowth of the infected cells in the population, as the samples were collected only 48 h post-infection.

**Table 2:** Hot spots of integration for viruses carrying the K258R IN mutation.

Genomic coordinates	Number of integrants
Chr14: 17749223–17757726	5
Chr13: 17630007–17635451	6
Chr21: 12443946–12448362	6
Chr21: 12534522–12533844	6
Chr13: 17669962–17678635	7
Chr14: 18151557–18159208	7
Chr22: 14673032–14672354	8
Chr1: 125173987–125183192	9
Chr22: 15024034–15019610	9
Chr1: 143246863–143248277	12

Hot spots are defined as 5+ integrations in a 10 kb window. Coordinates of hot spots and number of integrations by K258R mutant IN mapped to each hot-spot are shown.

**Fig. 3:**
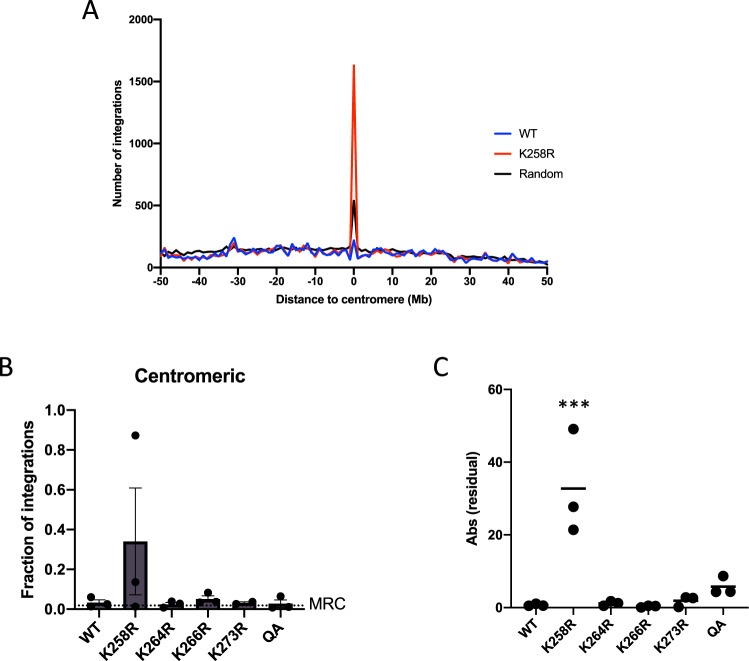
K258R mutant HIV-1 IN profoundly biases integration toward centromeres. **A** The distance to the nearest centromere was calculated for all WT and K258R mutant integration sites from three independent experiments. A 50 Mb window flanking each centromere was segmented into 100 equal sized bins of 1 Mb. The number of integrations falling in each bin was quantified and is shown as a count (WT in black, K258R in red). **B** Number of integrations located in centromeric regions were normalized to total detected integration sites and are shown as a percent of the total. Integration frequency into centromeres in the matched random control (MRC) data set is shown as a dashed line. Data is shown as the average of three independent biological replicates ± SEs. Statistical significance relative to MRC was calculated by one-way ANOVA corrected for multiple comparisons. **C** To assess variability of the altered integration centromere targeting phenotype we plotted the absolute residual from the mean for each independent trial. Statistical significance of variance was calculated using Levene’s test (***p < 0.0001, *n* = 3). Source data are provided as a Source Data file.

To better quantify all integration events in centromeric regions, we extracted genomic coordinates of centromeres from the hg38 human reference genome and determined the distance from each integration to the nearest centromere. In agreement with the hot-spot analysis, we found a dramatic increase in integration frequency in centromeric regions specifically for proviruses integrated by the K258R mutant IN as compared to WT (Table 1). Analyzing the cumulative data from pooling all the sequence libraries, we found approximately 8% of total K258R proviruses were integrated into centromeres, a far higher frequency than for the WT virus (tenfold more, a difference at high statistical significance, with Fisher’s exact *t* test, *p* < 0.0001). We detect <1% of the proviruses integrated by a WT IN in centromeres, even below what would be expected by random chance. The observed preference of K258R is specific for centromeric sequences, and we did not observe an increase in integration in the flanking peri-centromeric region (Fig. 3A).

We also analyzed the integration sites generated from the other acetylation mutant IN proteins. On average 1.7–4% of the detected proviruses integrated by these mutant IN proteins were detected in centromeric regions, a two to fourfold increase as compared to WT (Fig. 3B). It is of note that the quadruple acetylation mutant (K258/264/266/273R), though including the K258R mutation, did not produce the same dramatic phenotype as the K258R mutant, suggesting that the K258R mutation induces a distinctive gain-of-function phenotype not seen in the presence of the additional mutations. While all mutants exhibited a slight increase in preferential targeting to the centromeres, the K258R mutation alone strongly retargeted integration into centromeres at a strikingly high frequency, indicating that the effect of the K258R mutation in IN is unique to this residue, and not a general feature of blocking IN acetylation.

It should be noted that the magnitude of the observed phenotype in the NGS analysis was highly variable between independent replicate experiments. Cumulative data of all libraries yields highly significant differences in integration pattern between the K258R and WT viruses (Table S1), but examining individual experiments shows a wide range in the magnitude of the effect (Fig. 3B). The fraction of the mutant integrations in centromeres ranged from extraordinarily high (~80%—the vast majority of integrations) to very high (6%) to only moderately high (1%) in biological replicates. The variability is a genuine feature of the independent infections and not a feature of the PCR analysis, because repeat analysis of given infected cell DNA preparations gave closely matched results. To document this variability, we plotted the absolute value of the residuals from the mean observed in each replicate sequencing run for WT as well as in all IN mutants (Fig. 3C). The K258R mutation in IN produces a broad range of centromeric integration frequencies whereas WT IN and other IN mutant viruses gave a tight, uniform distribution around the mean in all trials. The potential for dramatically increased centromeric integration is a unique attribute of the K258R mutant IN.

The large variability of the observed integration targeting phenotype might be attributable to how repetitive DNAs are sequenced and/or mapped. Traditional bioinformatics tools to map sequence data to the genome are limited in their capacity to deal with repetitive sequences, and many repeat elements are not even present in genome assemblies because they cannot be accurately mapped. For this reason many integration site mapping studies to date focus exclusively on uniquely mapped reads to avoid the complexities of handling reads that map to multiple sites (“multi-mapping reads”). Our sequencing reads were mapped using a stringent Bowtie2 end-to-end alignment algorithm, with conservative reporting options that likely underestimate the true frequency of utilization of repetitive DNA as targets for integration. To obtain independent confirmation of the striking retargeting, we made use of several other bioinformatics tools commonly used in the field to re-analyze our integration site sequencing data.

We first confirmed the preference of the K258R mutant IN for integrating into centromeres using a Bowtie2-based sensitive local alignment strategy which allows for “soft-clipping” or omission of characters from the ends of reads in order to achieve the best alignment score. We further validated the centromeric integration preference of the K258R mutant IN using the BLAT mapping algorithm^34^, which is more commonly used amongst published integration site analysis studies. The BLAT mapping algorithm is based on BLAST and similarly reports all valid alignments above a set threshold score regardless of whether a read is unique or multi-mapping to repetitive sequences. Regardless of mapping algorithm, the data show that the K258R mutation in IN results in a dramatic redirection of integrations towards centromeres (Fig. S3). This site bias is not seen in any of the replicate tests of WT IN or other acetylation mutant IN proteins.

While the initial integration site mapping indicated that the K258R IN mutation induces a preference for integrating into centromeric regions, these algorithms do not identify specific target sequences and in fact do not even consider integration into the vast majority of repetitive sequences, which are largely excluded from the hg38 human reference genome. Centromeres are composed of tandem repeats, including both very short unit length repeats, and a high proportion of so-called alpha satellite sequence DNA comprised of alphoid repeat monomers with a unit length of approximately 171 bp^35^. A number of other satellite sequences are present at lower abundance in the centromeric regions as well^33,36,37^. To determine which class of repeats may be specifically targeted by the K258R mutant IN, sequencing reads were mapped directly to the RepeatMasker track from the UCSC Genome Browser^38^. The RepeatMasker track includes all known repetitive sequences present in the genome, including simple repeats and shorter repeat units that are not present in the reference genome assembly. This allowed us to quantify integrations into all known repetitive regions, both in the centromere and outside, as well as obtain information on the repeat classes that are preferred targets of integration.

**Fig. 4:**
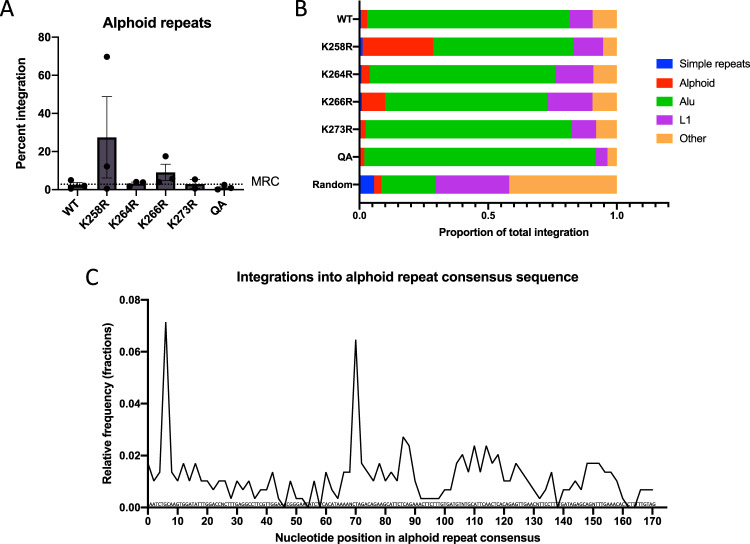
Mapping of proviral integrations to repetitive regions in the human genome. NGS reads from three independent biological replicate libraries were aligned to the RepeatMasker track from the UCSC genome browser. **A** The number of integrations mapping to alphoid DNA repeats was determined and normalized to the total number of mapped integrations and is shown as a percent of the total (*n* = 3, ±SEs). The frequency with which integrations would be expected to fall in alphoid repeats if integration were random is shown as a dashed line (MRC). **B** The proportion of integrations that mapped to specific repeat elements relative to the total number of reads that mapped to the RepeatMasker track is shown. Only the most commonly targeted repeat elements are displayed. **C** Schematic of integration sites along the length of a single alphoid repeat. Unique host sequences immediately flanking each integration by the K258R mutant IN were aligned to an alphoid repeat consensus sequence (AJ131208.1) using BLAST The consensus sequence was split into bins of two nucleotides and the number of integrations in each bin were counted. Shown are the integration counts falling in each bin summed over three replicates. Source data are provided as a Source Data file.

The K258R IN mutation causes a specific targeting of integrations into alphoid repeat sequences (Fig. 4A). This preference for alphoid repeats is not seen with WT IN or other acetylation mutant IN proteins, and indeed alpha satellite DNA specifically has been previously reported to be a disfavored target of WT HIV-1 integration^33^. The frequency of integrations into other common very-high abundance repeat classes such as Alu and L1 elements were not significantly different between WT and mutant INs (Fig. 4B). The K258R mutation of IN seems to uniquely redirect integrations to alpha satellite repetitive DNA and not other classes of repeat sequences.

For all integrations by the K258R mutant IN that mapped to the centromere, we extracted the immediate flanking host genome sequences (10 bp upstream and 10 bp downstream), removed all identical junctions to be conservative and then aligned these to the alphoid repeat consensus sequence (AJ131208.1). We observe selective sites of insertion within the alpha satellite sequence by the K258R mutant IN protein, with two preferred spots of integration at nucleotide position 6 and 69 in the alphoid consensus sequence (Figs. 4C, S4). The best alignment for the viral–host junction sequences at each hot spot was identical to the base position, but notably the flanking alphoid host sequences at all the junctions were distinct, and thus represented insertions into distinct members of the alphoid repeat family. These two preferred sites in the repeat do not share a high level of sequence identity, in either orientation. There are no known protein binding motifs near either of the preferred sites. It is thus unclear why either of these two sequences is a preferred hot spot for the mutant IN. Further, we observed integrations at nearly every nucleotide position within the consensus sequence indicating that the large number of insertions into the alpha repeats are truly independent integration events and not PCR amplification artifacts (Fig. S4).

**Fig. 5:**
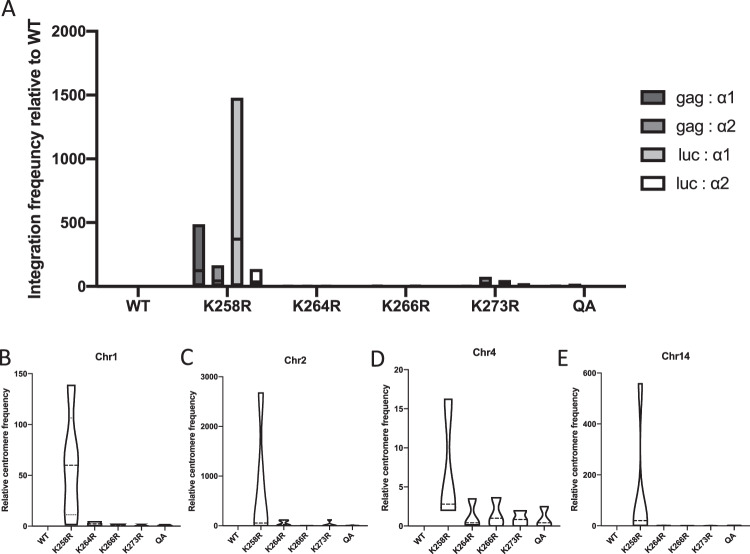
Quantification of integration frequency into centromeric regions by qPCR methods. **A** Integration into centromeric alphoid repeat DNA was quantified using a modified Alu-gag based nested PCR approach. Two unique primers were designed complementary to an alphoid repeat consensus sequence (α1, α2) and used instead of the typical Alu primer. Two primers at either end of the viral genome were used—either in the 5′ end of *gag* or in the 3′ UTR of the luciferase (*luc*) reporter gene. First nest PCR was performed with these four primer combinations. Shown are the results of a second nest quantitative PCR using LTR-specific primers normalized to total integrated provirus levels as measured by Alu-gag PCR. Data from a minimum of three independent replicates is shown relative to WT as box plots to show the minimum, maximum and mean values. **B**–**E** Quantitative PCR using chromosome-specific centromere primers. Viral LTR-host genome junctions were amplified and centromere content was subsequently quantified using qPCR with chromosome-specific primers (see Table S4 for all primer sequences) and normalized to total integrated provirus levels as measured by Alu-gag PCR. Shown is the relative centromere content for each infected sample relative to WT from a minimum of two independent biological replicates done in triplicate presented as a violin plot to accurately represent the data distribution. Source data are provided as a Source Data file.

Due to the variability in the magnitude of the phenotype as well as the limitations of deep sequencing and available analytic tools, we wanted to verify the observed altered integration site distribution using a second method. In a modification of the so-called “Alu-gag” method to quantify integration frequency, we devised a nested PCR approach to specifically assay for integrants in or near centromeric repeats. We replaced the primer located in the Alu repeat element that is typically used in Alu-gag assays with primers complementary to the alphoid repeat consensus sequence^33,39^. We tested two unique alphoid primers in our assay, and to analyze both the 5′ and 3′ ends of the provirus, we used primers complementary to either *gag* or luciferase respectively. This allowed for four unique combinations of primers in the first round of PCR that would selectively amplify proviruses in or near centromeric alphoid repeats. A subsequent second round quantitative PCR, using LTR-specific primers, reported the yield of amplified viral DNA. The assays, performed in independent infections, revealed a dramatic increase in the frequency of centromeric integration events for proviruses integrated by the K258R mutant IN confirming observations made by deep sequencing (Fig. 5A). The magnitude of the effect was again highly variable, both between primer combinations and within a given primer pair, but was always dramatic. The K258R mutant IN increased integration frequency near centromeric alphoid repeats over the wild-type control by an average of 30–400 fold. The alpha satellite bias was again only seen with the K258R mutant. All other mutations blocking other acetylation sites displayed a similar level of centromeric integrations as wild-type controls.

All the data shown above report on infections of HeLa cells, used because of their high susceptibility to infection and the availability of good sequence databases. To see if the centromeric targeting occurred in another cell line, and one more relevant to HIV-1 replication in vivo, we repeated the infections and alphoid-virus PCR assays with mutant and wild-type virus preparations in the Jurkat T-cell line. In three biological replicates, using independent infections, we again found higher frequencies of integrations near alpha repeats for the K258R mutant compared to the wild-type (Fig. S5). As with HeLa cells, the frequencies were highly variable from experiment to experiment and with the choice of primer, but were always higher for the mutant over the wild-type control and often dramatically so. These results indicate that the phenotype of the K258R mutant is not restricted to a single cell line but may be manifest in many cell types, including lymphoid T cells.

In our initial analysis to identify common sites of integration from NGS data, the identified genomic hot spots were all found in only a subset of chromosomes (Table 2). To determine whether the K258R mutant IN displayed any particular chromosomal preference, we also performed a qPCR assay utilizing chromosome-specific non-repetitive centromere primers to quantify specific centromeric DNA content present at the LTR-host genome junction. Shown are some representative examples using chromosome-specific primers for chromosomes 1, 2, 4, and 14 (Fig. 5B–E). The K258R mutant virus was observed to integrate much more frequently than WT or any other acetylation IN mutant viruses at sites near the centromeres regardless of chromosome. The apparent bias for some chromosomes that we observed in the initial “hot spot” analysis of the NGS data could be due to gaps or discrepancies in the assignments of the centromere sequences present in the genome assembly database. The PCR data suggest that K258R virus is targeted to centromeres of many, if not all, chromosomes.

**Fig. 6:**
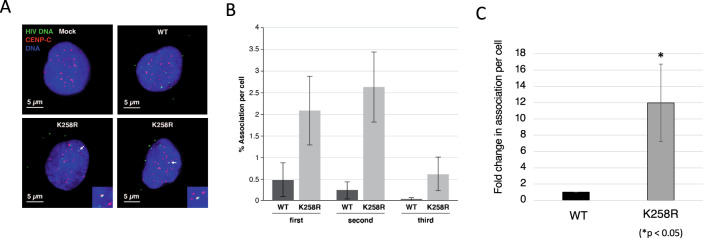
Enrichment of K258R IN mutant HIV-1 in centromeres. HeLa cells were infected with WT and K258R for 16 h at MOI 10. Infected cells were stained for CENP-C and then subjected to HIV-1 DNA FISH. **A** Representative images of the association between CENP-C (red) and HIV-1 DNA (green). The association indicated by arrows was represented in an inset. Scale bars; 5 μm. **B** Quantification of HIV-1 DNA signals co-localizing with CENP-C. HIV-1 DNA signals detected in the nucleus were counted for the colocalization with CENP-C in every single z-section of each nucleus. An average of 50 nuclei was analyzed in each experiment. The average percent association per cell is shown for each of three biological replicates (±SEs; *n* = approximately 50 for each bar). **C** Fold change in K258R association with CENP-C over WT. Data is average ± SEs of three independent experiments. Statistical significance was determined by two-tailed Student *t* test. *p < 0.05. Source data are provided as a Source Data file.

To confirm that the K258R mutant IN is retargeting integrations to the centromere using a non-PCR based method, we made use of a fully orthogonal readout, utilizing simultaneous fluorescent in situ hybridization (FISH) of HIV-1 DNA and immunostaining of centromeres. HIV-1 DNA was visualized by FISH 16 h after infection under deconvolution microscopy. Centromere associated protein, CENP-C, was used as a marker for centromeres (Fig. 6A). To enumerate HIV-1 DNA colocalization with CENP-C, z-sections of nuclear volumes were obtained and association of the two signals was scored. In this assay we observed that viral DNA from the K258R mutant IN exhibited a 4–20-fold increase in CENP-C association on average relative to viral DNA from virus with WT IN (Fig. 6B; average 12-fold). HIV-1 DNA foci were not detected in control cells treated with AZT (data not shown). These microscopy data obtained very early after infection are in support of the observation of increased integration in centromeric regions versus selective outgrowth of cells containing centromeric integrations.

Because HIV-1 integration is in part targeted through host factor interactions, it is plausible that the K258R mutation in the IN protein could modulate integration site selection by mediating differential binding of a specific host factor. To test this possibility, we generated mammalian expression vectors expressing either WT or K258R mutant IN protein and tested for host binding proteins. In both cases, the IN protein was N-terminally tagged with HA for immunoprecipitation. HEK293T cells were transfected with the IN plasmids and lysates were harvested after 24 h of expression. Adequate and comparable expression of both WT and mutant IN proteins was confirmed via western blot using both HA- and IN-specific antibodies. WT and mutant IN were immunoprecipitated and interacting host proteins were subject to mass spectrometry for identification.

We identified 43 and 56 proteins that bound to WT or K258R IN proteins, respectively, above the background of an empty vector control (Table S2). The majority of these host factors were shared between WT and K258R mutant IN. Based on a preliminary gene ontology analysis, the majority of factors that bind either WT or mutant IN protein are generic nucleic acid binding proteins (Table S3). Approximately a third of the 43 proteins we detected binding to WT IN have been previously reported by another mass spectrometry screen done in HEK293T cells, validating our approach^40^. We did not detect LEDGF in our immunoprecipitation, which is not unexpected due to its low abundance and poor solubility^40^. However, because it is known to bind to the core domain of HIV-1 IN (W131, I161, R166, Q168, Q170) we expect LEDGF binding would not be perturbed by the K258R mutation in the IN protein^41^.

Mutant K258R IN bound to the majority of previously reported host factors, but several binding partners were identified with enhanced binding to the K258R mutant IN. Two factors involved in mitotic chromosome condensation (NCAPD3 and SMC4) along with multiple components of the catalytic core of the protein phosphatase I (PPI) complex were enriched in the MS screen of the factors bound with the K258R mutant IN as compared to WT. These factors have links to heterochromatin formation and regulation at the centromere as well as in chromatin condensation during mitosis^42–45^. The binding of these factors to IN was examined directly by expressing tagged mutant and WT IN in HeLa cells, recovering the IN proteins by IP, and assaying the bound proteins for these factors with Western blots probed with specific antibodies. All four proteins bound avidly to both tagged INs, but with no apparent preference for the mutant IN seen in the MS data (Fig. S6A). Thus, these binding data do not reveal a basis for the selectivity of the integration phenotype for K258R. Treating the lysates with benzonase to remove nucleic acids did not have dramatic effects on the coIP of the proteins with IN, though there was a slightly increased loss of SMC4 and NCAPD3 from the K258R mutant IN (Fig. S6B). It is possible that the in vitro binding assays do not detect differences in binding in vivo. It is also possible, of course, that the targeting activity of the factors, once bound, are affected by the IN mutation and that this is not reflected in the coIPs. The functional importance of any of these factors in targeting remains to be examined. In addition, gene ontology analysis of the partners revealed an enrichment for genes involved in tRNA processing as well as antiviral interferon stimulated genes (Table S3). It is not immediately obvious how preferential binding of the mutant IN to these proteins would redirect integrations to centromeric regions.

To our knowledge, the K258R mutant shows the most dramatic retargeting of integration sites induced by an IN mutation reported for any retrovirus so far. Similar cases of strong retargeting can be found amongst yeast retrotransposons, many of which exhibit highly preferential integration. Integration preferences amongst the yeast Ty elements are largely due to the binding of the IN protein to host proteins which serve as tethering factors. Preferential targeting to heterochromatin by yeast Ty5 elements is achieved by IN binding to Sir4, and blocking IN-Sir4 binding leads to a large scale redirection of integrations throughout the genome^24,46^. More recently, it was found that when binding of the Ty1 IN protein to its tethering factor AC40 was blocked, integrations were strikingly retargeted away from PolI and PolIII-transcribed genes to subtelomeres^47^. However, retroviruses have less strongly biased integration preferences and retargeting effects of previous mutations in IN have been more subtle. HIV-1 integration does make critical use of host factors, and knockout of the main integrase tethering factor, LEDGF, has a large impact on overall integration efficiency and virus replication, but this results in a less than twofold redirection phenotype for the residual integration events^7,13,14,48^. The most striking retargeting of a retrovirus thus far observed has been seen when a point mutation is introduced into the Gag protein of PFV^19^. Interestingly, this mutation also results in a retargeting of PFV integration to centromeres. That mutations in the IN protein of HIV-1 or the Gag protein of PFV can generate similar phenotypes raises the possibility that centromeric integration may be a conserved default preference that is actively avoided by the use of host tethering factors to locate more optimal integration sites for viral expression.

The redirection of integrations to the centromere caused by the K258R mutation in the IN protein is especially provocative in light of recent work linking centromeric HIV-1 integrations to viral latency and control. Proviruses in centromeric satellite DNA have been found in the latent reservoir of patients as well as associated with deep viral latency in past reactivation studies^39,49,50^. Thus, integration into these “gene deserts” promotes viral silencing and the formation of the major impediment to HIV-1 cure. More recently it has been shown that proviral sequences from elite controllers were also preferentially enriched in centromeric satellite DNA^51^, suggesting that a common process may underlie the resultant proviral silencing in both settings. We have found the K258R mutation present at very low frequency in proviral sequence repositories of latent proviruses, drug resistant mutants, and from patients on suppressive antiretroviral therapy but the extent of the enrichment of this mutation in these sequence pools is unclear due to the paucity of sequence information available^52–57^. Understanding how this single point mutation can cause such a striking retargeting of integration will be important for characterizing and ultimately manipulating the mechanisms that underlie viral latency and long term control in patients.

## Methods

### Cells and plasmids

HEK293T (ATCC CRL-3216), Jurkat (ATCC TIB-152) and HeLa (ATCC CCL-2) cells were cultured in DMEM media supplemented with 10% FBS and 1% pen-strep at 37 °C, 5% CO_2_.

HIV-1 viral constructs were derived from the replication defective pNL4.3R-E- plasmid (NIH AIDS Reagent Program #3148) carrying a firefly luciferase reporter gene in the *nef* open reading frame. Mutations were introduced into the IN open reading frame using PCR site-directed mutagenesis with custom primer^29^.

### Transfection, virus preparation and infection

To prepare pseudotyped virus for infection, HEK293T cells were co-transfected with the pNL4.3.Luc.R-E- viral vector as well as a plasmid expressing the vesicular stomatitis virus glycoprotein (VSV-G) envelope (pMD2.G) using Lipofectamine 3000 (Life Technologies) according to basic manufacturer’s protocol. Viral supernatants were collected at 48 h post-transfection, filtered through a 0.45 micron filter, and DNase treated to eliminate plasmid DNA contamination. Viral preparations were normalized by RNA viral genome content, diluted 3-fold with fresh culture medium and immediately used for infection of HeLa cells. The preparations yielded an approximate multiplicity of infection (MOI) between 0.3 and 1).

### Luciferase assay

Successful viral transduction was assayed after 48 h by measuring luciferase activity with the Promega Luciferase Assay System (Cat# E4550). Luminescence (RLU) measurements were normalized for total cell count as determined by protein concentration.

### Quantitative PCR for viral DNA intermediate and RNA analysis

DNA was isolated from acutely infected cells 2 days post-infection using the Qiagen DNeasy Blood and Tissue Kit. Quantitative PCR for viral DNAs was performed using FastStart Universal SYBR Green Mastermix (Bio-Rad) according to manufacturer’s protocol on ABI 7500 Fast Real Time PCR System. Total viral DNA was quantified using primers complementary to the luciferase gene. RT products were detected with LTR-specific primers. 2-LTR circles were quantified as previously published and normalized to total virus^58^. Integrated proviruses were quantified using the published Alu-gag nested PCR protocol^59,60^.

To quantify steady state viral mRNA levels, RNA was extracted from cells using a standard Trizol protocol. RT was performed using random hexamer primers with Maxima H Reverse Transcriptase (Thermo Fisher). Viral cDNA was then quantified via qPCR using primers complementary to spliced *tat* message and normalized to a housekeeping gene.

All primers used for quantification can be found in Table S4. A minimum of three biological replicates were performed per experiment with technical duplicates within each experiment for precision. Biological replicates refer to completely independent experiments, while technical replicates refer to repeated measures of the same samples. A single factor ANOVA analysis was used to identify significant changes (*p* < 0.05). If appropriate, pairwise comparisons were performed using a two-tailed paired *t* test assuming unequal variance.

### Next generation sequencing (NGS) library construction

DNA sequencing libraries were prepared as described previously^29,32,61^. Briefly, five micrograms of purified genomic DNA from infected cells was randomly sheared using a Branson 450 Digital Sonifier. Sheared ends of DNA were subsequently repaired, A-tailed and ligated to custom oligonucleotide adaptors. Nested PCR was performed using viral and adaptor specific primers to enrich the library for proviral-host genome junctions and add necessary index and flow cell attachment sequences for Illumina (see Table S5 for library adaptor and primer sequences). PCRs were performed such that the final library product should contain 40 bp of the 3′ viral LTR sequence immediately prior to the junction with the host genome sequence. Sequencing was performed using the Illumina MiSeq platform with 300 bp read length. Three unique biological replicate libraries were generated and sequenced independently.

### Integration site mapping data analysis

Reads were initially demultiplexed by unique dual barcodes and filtered to exclude reads not containing an initial viral LTR sequence at the host junction using a custom python script^29^. We required an exact match to the terminal 40 nt of the 3’ viral LTR. All reads were then trimmed to remove both leading viral sequence as well as any residual adaptor sequences. Reads of less than 20 nucleotides after all filtering steps were discarded. Remaining reads were mapped to the GRCh38 human genome using either Bowtie2 or BLAT^34,62^.

For majority of analyses, unless otherwise noted, reads were first aligned to the pNL4.3.Luc.R-E- vector genome to remove any viral auto-integration or circular products. The remaining reads were then aligned to the unmasked GRCh38 human reference genome using Bowtie2 end-to-end alignment with a seed length of 28 nucleotides and a maximum of 2 mismatches permitted in the seed. Reads that mapped to multiple locations were not suppressed. Instead, best alignment was reported. For reads with equally good alignments, one of the alignments was reported at random.

Where noted, sequences were further locally aligned to the unmasked GRCh38 genome build using either Bowtie2 sensitive local settings or BLAT. For BLAT analysis, alignments were filtered for 95% minimum identity and a minimum score of 30. All acceptable alignments above this threshold were reported with scores based on number of matched/mismatched bases and a default gap penalty. For reads mapping to multiple locations equally well, all alignments were reported. Parameters for Bowte2 local mapping were 20 nt seed length, allowing 0 mismatches in the seed.

Reads were also aligned directly to the RepeatMasker genome track from UCSC using the same mapping algorithms. Only data from Bowtie2 local mapping is shown here. The RepeatMasker track contains all annotated repeat sequences in the human genome^38^. Number of integrations falling into each specified repeat class was calculated and presented as a percent of the total number of integrations mapped.

### Hot-spot analysis of viral integrations

Using a previously reported custom perl script, common sites, or “hot spots”, of viral integration were determined^63,64^. First, identical reads, or PCR duplicates were condensed. Second, reads with identical junctions but varying sonication breakpoints were condensed to eliminate any confounding effects of clonal expansion. To be stringent, reads with highly similar sequences (i.e., >95% identity) were also combined to eliminate any artifacts produced from small PCR or sequencing errors. From here, “hot spots” of viral integration were determined using a sliding window approach^30^. This script searches for multiple integrations falling within a set range of nucleotides from each other. For this study “hot spots” were defined as regions of 10 kb or less with five or more unique viral integrations.

### Analysis of integration sites with respect to genomic annotations

Genomic coordinates of annotated RefSeq genes, transcription start sites, CpG islands and DNase hypersensitivity regions were extracted from the GRCh38 genome assembly via the UCSC Genome Browser. The genomic coordinates of centromeric sequences were also extracted from UCSC Genome Browser. Locations of RNA polymerase II binding sites and histone modifications were extracted from ENCODE data sets generated from uninfected HeLa cells (Pol II: ENCFF246QVY; H3K27Ac: ENCFF113QJM; H3K9me3: ENCFF712ATO; H3K36me3: ENCFF864ZXP; H3K4me3: ENCFF862LUQ). Distance of proviral integrations to nearest feature was calculated using BedTools^65^. A MRC data set of comparable size was generated with BedTools Random command and mapped in parallel to experimental data sets.

A one-sample *t* test was used to compare integration distribution between experimental samples and MRC (Table S6). To gauge the statistical significance of differences in integration patterns between WT IN and mutant IN we used a paired, two-tailed *t* test of three independent replicate data sets for each condition or Fisher’s exact test on the aggregate integration data (Table S1).

Consensus sequence at the sites of integration was determined by MEME software suite^66^.

### Sequence analysis of centromeric integration sites

The host sequence flanking the site of integration was extracted from Bed coordinates of mapped integration sites. Only integration events into alphoid repeats and/or centromeres were used for this analysis. To align sites of integration along the repeat length of the alphoid repeat, we used only the ten base pairs flanking the site of integration (total length 20 bp) to align to a consensus sequence for the alphoid repeat monomer (AJ131208.1). Only unique junctions were aligned. Alignments were performed with BLAST^67^. For count purposes, we defined 85 bins spanning the alphoid repeat monomer, each consisting of two base pairs, and counted the number of integrations falling in each bin.

### PCR assays for quantifying centromeric integrations

To determine if centromeric DNA sequences were over-represented in library preparations, we made use of previously reported unique chromosome-specific centromere primers^68^. Amplified viral–host genome fragments from library preparations were used in a qPCR assay using centromere specific primers to relatively compare quantities of centromeric DNA sequences between infections with viruses carrying WT or mutant IN proteins.

To look more generically at integration into all centromeres, we devised a nested PCR assay based on both the basic Alu-gag PCR protocol for quantifying proviral integration and a previously published assay using alpha satellite specific primers (alphoid-1, alphoid-2)^33^. For the first nest, one of two primers complementary to the alpha satellite consensus sequence were used in conjunction with either a 5′ viral specific primer (5′-*gag*) or a 3′ viral specific primer (3′-luc). First round PCRs were performed with standard Taq polymerase for 20 cycles with a 5 min elongation time and an annealing temperature of either 52 °C or 58 °C for alphoid-1 or alphoid-2 primers respectively. For validation purposes, a number of randomly selected fragments were cloned from the first rounds of PCR and sequenced by Sanger sequencing to verify that we were indeed amplifying alphoid repeats at the viral–host genome junction. LTR-specific primers were then used for the second nest quantitative PCR. These values were normalized to total LTR content in original unamplified DNA (Jurkat cells) or integrated provirus as measured by Alu-gag PCR (HeLa cells). See Table S4 for primer sequences used.

### Fluorescent in situ hybridization (FISH) assays

HeLa cells were grown on 12 mm circle coverslips (Electron Microscopy Sciences) for 24 h, exposed to mock supernatants or those containing WT or K258R IN mutant HIV-1 at an MOI of 10 for 16 h, fixed with 4% PFA for 10 min at room temperature (RT), and permeabilized with 0.5% Triton X-100 for 10 min. Cells were incubated with the primary antibody for CENP-C (PA5-66154, 1:100, Invitrogen) for 1 h at RT and with goat anti-rabbit IgG (H + L) secondary antibody (1:2000, Invitrogen) for 30 min at RT, fixed with 2% PFA, and then treated with RNase (0.1 mg/ml, Qiagen) for 1 h at 37 °C. Cells were permeabilized with ice-cold 0.5% Triton X-100/0.1 M HCL for 10 min on ice. These cells were incubated in 2 × SSC for 30 min at 70 °C, 0.1 × SSC for 1 min at RT, 0.07 N NaOH for 1 min at RT. Then, cells were equilibrated in ice-cold 0.1 × SSC for 1 min at 4 °C, ice-cold 2 × SSC for 1 min at 4 °C, dehydrated in ethanol (70%, 85%, and 100% ethanol for 2 min at RT), and air-dried. For centromere FISH, 1 μl of centromere probes to either chromosome 8 or 12 (Empire Genomics) were incubated with cells grown on 12 mm circle coverslips for 48 h at 37 °C. After hybridization, cells were washed with 2 × SSC for 30 min at 37 °C, 2 × SSC for 30 min at RT, 1 × SSC for 30 min at RT, and stained with Hoechst 33258 (1 mg/ml). For HIV-1 DNA FISH, HIV-1 DNA probes were generated as a pool of 48 distinct 20-nucleotide long oligonucleotides specific to the integrase gene of HIV-1 WT pNL4.3 plasmid and labeled with Atto-565 dye (Biomers). 20 mM of HIV-1 DNA probes were used per 12 mm circle coverslips. Coverslips were mounted onto glass slides using Gel Mount (Electron Microscopy Sciences) containing an anti-fade reagent. Dried slides were imaged on a Deltavision Core microscope system fitted with an automated stage (Leica Microsystems) and images were captured in z-series on a CCD digital camera. Out-of-focus light was digitally removed using the Softworks deconvolution software (Leica Microsystems). To examine the colocalization of centromeres and CENP-C, 3-D volume projections were generated using the Softworx analysis program. Images were exported as tif files. The association between CENP-C and HIV-1 DNA was analyzed in a single z-section level and confirmed using the Softworks 3-D volume viewer software (Leica Microsystems).

### Co-immunoprecipitation of IN proteins and mass spectrometry

Either WT HIV-1 IN or IN harboring the K258R mutation was cloned into a mammalian expression vector (pJET). Both proteins had an N-terminal HA-tag for immunoprecipitation. As a negative control, we also transfected cells with an empty HA-vector. Constructs were transfected into HEK293T cells as described. After 24 h, cells were collected, washed and lysed with an NP-40 lysis buffer (20 mM Tris HCl, pH 8; 137 mM NaCl, 2 mM EDTA and 1% NP-40). Adequate, comparable expression of WT and mutant IN proteins was confirmed via Western blot using HA-specific or IN-specific antibodies.

Cell lysates were subsequently mixed with BSA blocked HA-coated magnetic beads (Pierce) and rotated overnight at 4 °C. Beads were washed three times with lysis buffer, finished with two PBS washes and sent for mass spectrometry analysis (Rockefeller Mass Spectrometry Core Facility). The mass spectrometry proteomics data have been deposited to the ProteomeXchange Consortium via the PRIDE partner repository^69–71^ with the data set identifier PXD031825.

MS results were filtered by number of peptides detected vs. an empty HA-vector control. Only proteins with five or more spectral counts were considered. Proteins were considered enriched when there was a minimum of 5-fold more unique spectral counts detected in the IN immunoprecipitation vs. the control precipitation. Enriched peptides immunoprecipitated by WT and K258R mutant IN were further subjected to gene ontology analysis performed with gProfiler software^72^.

Binding of K258R IN to selected protein binding partners was confirmed by co-immunoprecipitation monitored by Western blot. 293T cells were transfected to express HA-tagged versions of WT or mutant IN, and lysed with RIPA buffer after 24 h. The IN proteins were recovered by IP as described above and the bound proteins were analyzed by SDS PAGE and Western blotting with specific antisera (SMC4: Abcam ab17958, NCAPD3: Abcam ab70349, PPP1CB: Abcam ab53315, PPP1CC: Bethyl A300-906A, HA: Biolegend 901501, GAPDH: Millipore-Sigma CB1001). Blots were washed three times with 1× TBST for 10 min each and developed with GE ECL Western Blotting Detection System (RPN2106).

### Reporting summary

Further information on research design is available in the Nature Research Reporting Summary linked to this article.

## Supplementary information

Supplementary Information

Reporting Summary

## Data Availability

Sequencing reads generated as part of this study are available at the NCBI Sequencing Read Archive: (accession code SRP322500). The mass spectrometry proteomics data have been deposited to the ProteomeXchange Consortium via the PRIDE partner repository with the data set identifier PXD031825. Source data are provided with this paper.

## Source data

Source Data

## Code availability

Code uniquely generated for this analysis is available upon request.

## References

[1] Bowerman B, Brown PO, Bishop JM, Varmus HE (1989). A nucleoprotein complex mediates the integration of retroviral DNA. Genes Dev..

[2] Brown PO, Bowerman B, Varmus HE, Bishop JM (1989). Retroviral integration: structure of the initial covalent product and its precursor, and a role for the viral IN protein. Proc. Natl Acad. Sci. U.S.A..

[3] Mitchell RS (2004). Retroviral DNA integration: ASLV, HIV, and MLV show distinct target site preferences. PLoS Biol..

[4] Debyser Z, Christ F, De Rijck J, Gijsbers R (2015). Host factors for retroviral integration site selection. Trends Biochem. Sci..

[5] Schröder ARW (2002). HIV-1 integration in the human genome favors active genes and local hotspots. Cell.

[6] Ciuffi A (2005). A role for LEDGF/p75 in targeting HIV DNA integration. Nat. Med..

[7] Llano M (2006). An essential role for LEDGF/p75 in HIV integration. Science.

[8] Maertens G (2003). LEDGF/p75 is essential for nuclear and chromosomal targeting of HIV-1 integrase in human cells. J. Biol. Chem..

[9] McNeely M (2011). In vitro DNA tethering of HIV-1 integrase by the transcriptional coactivator LEDGF/p75. J. Mol. Biol..

[10] Sharma A (2013). BET proteins promote efficient murine leukemia virus integration at transcription start sites. Proc. Natl Acad. Sci. U.S.A..

[11] De Rijck J (2013). The BET family of proteins targets moloney murine leukemia virus integration near transcription start sites. Cell Rep..

[12] Kvaratskhelia M, Sharma A, Larue RC, Serrao E, Engelman A (2014). Molecular mechanisms of retroviral integration site selection. Nucleic Acids Res..

[13] Marshall, H. M. et al. Role of PSIP 1/LEDGF/p75 in lentiviral infectivity and integration targeting. *PLoS ONE*10.1371/journal.pone.0001340 (2007). 10.1371/journal.pone.0001340 PMC2129110 18092005

[14] Shun M-C (2007). LEDGF/p75 functions downstream from preintegration complex formation to effect gene-specific HIV-1 integration. Genes Dev..

[15] Lesbats P, Parissi V (2018). Retroviral integration site selection: a running Gag?. Microb. Cell.

[16] Fassati A (2012). Multiple roles of the capsid protein in the early steps of HIV-1 infection. Virus Res..

[17] Novikova M, Zhang Y, Freed EO, Peng K (2019). Multiple roles of HIV-1 capsid during the virus replication cycle. Virol. Sin..

[18] Sowd GA (2016). A critical role for alternative polyadenylation factor CPSF6 in targeting HIV-1 integration to transcriptionally active chromatin. Proc. Natl Acad. Sci. U.S.A..

[19] Lesbats P (2017). Structural basis for spumavirus GAG tethering to chromatin. Proc. Natl Acad. Sci. U.S.A..

[20] Wanaguru M, Barry DJ, Benton DJ, O’Reilly NJ, Bishop KN (2018). Murine leukemia virus p12 tethers the capsid-containing pre-integration complex to chromatin by binding directly to host nucleosomes in mitosis. PLoS Pathog.

[21] A B, P L (2021). Light and shadow on the mechanisms of integration site selection in yeast Ty retrotransposon families. Curr. Genet..

[22] Zou S, Ke N, Kim JM, Voytas DF (1996). The Saccharomyces retrotransposon Ty5 integrates preferentially into regions of silent chromatin at the telomeres and mating loci. Genes Dev..

[23] Xie W (2001). Targeting of the yeast Ty5 retrotransposon to silent chromatin is mediated by interactions between integrase and Sir4p. Mol. Cell. Biol..

[24] Dai J, Xie W, Brady TL, Gao J, Voytas DF (2007). Phosphorylation regulates integration of the yeast Ty5 retrotransposon into heterochromatin. Mol. Cell.

[25] Andrake MD, Skalka AM (2015). Retroviral integrase: then and now. Annu. Rev. Virol..

[26] Chen L, Keppler OT, Schölz C (2018). Post-translational modification-based regulation of HIV replication. Front. Microbiol..

[27] Cereseto A (2005). Acetylation of HIV-1 integrase by p300 regulates viral integration. EMBO J..

[28] Terreni M (2010). GCN5-dependent acetylation of HIV-1 integrase enhances viral integration. Retrovirology.

[29] Winans S, Goff SP (2020). Mutations altering acetylated residues in the CTD of HIV-1 integrase cause defects in proviral transcription at early times after integration of viral DNA. PLOS Pathog..

[30] Berry CC, Ocwieja KE, Malani N, Bushman FD (2014). Comparing DNA integration site clusters with scan statistics. Bioinformatics.

[31] Justice J (2015). The MET gene is a common integration target in avian leukosis virus subgroup J-induced chicken hemangiomas. J. Virol..

[32] Malhotra S (2017). Selection for avian leukosis virus integration sites determines the clonal progression of B-cell lymphomas. PLoS Pathog.

[33] Carteau S, Hoffmann C, Bushman F (1998). Chromosome structure and human immunodeficiency virus type 1 cDNA integration: centromeric alphoid repeats are a disfavored target. J. Virol..

[34] Kent WJ (2002). BLAT—the BLAST—like alignment tool. Genome Res..

[35] McNulty SM, Sullivan BA (2018). Alpha satellite DNA biology: finding function in the recesses of the genome. Chromosome Res..

[36] Miga KH (2019). Centromeric satellite DNAs: hidden sequence variation in the human population. Genes.

[37] Hartley G, O’neill RJ (2019). Centromere repeats: hidden gems of the genome. Genes.

[38] Tarailo-Graovac, M. & Chen, N. Using RepeatMasker to identify repetitive elements in genomic sequences. *Curr. Protoc. Bioinform.*10.1002/0471250953.bi0410s25 (2009). 10.1002/0471250953.bi0410s25 19274634

[39] Jordan A, Bisgrove D, Verdin E (2003). HIV reproducibly establishes a latent infection after acute infection of T cells in vitro. EMBO J..

[40] Jäger S (2012). Global landscape of HIV-human protein complexes. Nature.

[41] Cherepanov P, Ambrosio ALB, Rahman S, Ellenberger T, Engelman A (2005). Structural basis for the recognition between HIV-1 integrase and transcriptional coactivator p75. Proc. Natl Acad. Sci. U.S.A..

[42] Wang J (2017). Mutation of Arabidopsis SMC4 identifies condensin as a corepressor of pericentromeric transposons and conditionally expressed genes. Genes Dev..

[43] Samoshkin, A. et al. Human condensin function is essential for centromeric chromatin assembly and proper sister kinetochore orientation. *PLoS ONE* **4**, e6831 (2009). 10.1371/journal.pone.0006831 PMC2730017 19714251

[44] Leonard J (2015). Condensin relocalization from centromeres to chromosome arms promotes Top2 recruitment during anaphase. Cell Rep..

[45] de Castro IJ (2017). Repo-Man/PP1 regulates heterochromatin formation in interphase. Nat. Commun..

[46] Xie W (2001). Targeting of the yeast Ty5 retrotransposon to silent chromatin is mediated by interactions between integrase and Sir4p. Mol. Cell. Biol..

[47] Asif‐Laidin, A. et al. A small targeting domain in Ty1 integrase is sufficient to direct retrotransposon integration upstream of tRNA genes. *EMBO J*. 10.15252/embj.2019104337 (2020). 10.15252/embj.2019104337 PMC7459421 32677087

[48] Fadel HJ (2014). TALEN knockout of the PSIP1 gene in human cells: analyses of HIV-1 replication and allosteric integrase inhibitor mechanism. J. Virol..

[49] Lewinski MK (2006). Retroviral DNA integration: viral and cellular determinants of target-site selection. PLoS Pathog..

[50] Cohn, L. B. et al. HIV-1 integration landscape during latent and active infection. *Cell*10.1016/j.cell.2015.01.020 (2015). 10.1016/j.cell.2015.01.020 PMC4371550 25635456

[51] Jiang, C. et al. Distinct viral reservoirs in individuals with spontaneous control of HIV-1. *Nature*10.1038/s41586-020-2651-8 (2020). 10.1038/s41586-020-2651-8 PMC7837306 32848246

[52] Foley, B. et al. HIV sequence compendium 2018, Technical Report LA-UR-18-25673. 10.2172/1458915.

[53] Gatanaga, H. et al. Drug-resistant HIV-1 prevalence in patients newly diagnosed with HIV/AIDS in Japan. *Antiviral Res*. 10.1016/j.antiviral.2006.11.012 (2007). 10.1016/j.antiviral.2006.11.012 17194486

[54] Hattori, J. et al. Trends in transmitted drug-resistant HIV-1 and demographic characteristics of newly diagnosed patients: nationwide surveillance from 2003 to 2008 in Japan. *Antiviral Res*. 10.1016/j.antiviral.2010.07.008 (2010). 10.1016/j.antiviral.2010.07.008 20692295

[55] Gondim, M. V. P. et al. Heightened resistance to host type 1 interferons characterizes HIV-1 at transmission and after antiretroviral therapy interruption. *Sci. Transl. Med*. 10.1126/SCITRANSLMED.ABD8179 (2021). 10.1126/scitranslmed.abd8179 PMC7923595 33441429

[56] Margot, N. A., Ram, R. R., White, K. L., Abram, M. E. & Callebaut, C. Antiviral activity of HIV-1 integrase strand-transfer inhibitors against mutants with integrase resistance-associated mutations and their frequency in treatment-naïve individuals. *J. Med. Virol*. 10.1002/jmv.25564 (2019). 10.1002/jmv.25564 31389026

[57] Kuo, H. H. et al. Blood and lymph node dissemination of clonal genome-intact human immunodeficiency virus 1 DNA sequences during suppressive antiretroviral therapy. *J. Infect. Dis*. 10.1093/infdis/jiaa137 (2020). 10.1093/infdis/jiaa137 PMC8493664 32236405

[58] Mandal D, Prasad VR (2009). Analysis of 2-LTR circle junctions of viral DNA in infected cells. Methods Mol. Biol..

[59] Butler SL, Hansen MS, Bushman FD (2001). A quantitative assay for HIV DNA integration in vivo. Nat. Med..

[60] O’Doherty U, Swiggard WJ, Jeyakumar D, McGain D, Malim MH (2002). A sensitive, quantitative assay for human immunodeficiency virus type 1 integration. J. Virol..

[61] Serrao, E., Cherepanov, P. & Engelman, A. N. Amplification, next-generation sequencing, and genomic DNA mapping of retroviral integration sites. *J. Vis. Exp*. 2016 **109**, 53840 (2016). 10.3791/53840 PMC4829050 27023428

[62] Langmead B, Trapnell C, Pop M, Salzberg SL (2009). Ultrafast and memory-efficient alignment of short DNA sequences to the human genome. Genome Biol..

[63] Justice JF, Morgan RW, Beemon KL (2015). Common Viral Integration Sites Identified in Avian Leukosis Virus-Induced B-Cell Lymphomas. MBio.

[64] Malhotra S (2017). Selection for avian leukosis virus integration sites determines the clonal progression of B-cell lymphomas. PLOS Pathog..

[65] Quinlan AR, Hall IM (2010). BEDTools: a flexible suite of utilities for comparing genomic features. Bioinformatics.

[66] Bailey, T. L. et al. MEME Suite: Tools for motif discovery and searching. *Nucleic Acids Res*. **37**, W202-8 (2009). 10.1093/nar/gkp335 PMC2703892 19458158

[67] Altschul, S. F., Gish, W., Miller, W., Myers, E. W. & Lipman, D. J. Basic local alignment search tool. *J. Mol. Biol*. 10.1016/S0022-2836(05)80360-2 (1990). 10.1016/S0022-2836(05)80360-2 2231712

[68] Contreras-Galindo R (2017). Rapid molecular assays to study human centromere genomics. Genome Res..

[69] Perez-Riverol Y (2022). The PRIDE database resources in 2022: a hub for mass spectrometry-based proteomics evidences. Nucleic Acids Res..

[70] Perez-Riverol Y (2016). PRIDE inspector toolsuite: moving toward a universal visualization tool for proteomics data standard formats and quality assessment of proteomexchange datasets. Mol. Cell. Proteom..

[71] Deutsch EW (2020). The ProteomeXchange consortium in 2020: enabling ‘big data’ approaches in proteomics. Nucleic Acids Res..

[72] Reimand J, Kull M, Peterson H, Hansen J, Vilo J (2007). g:Profiler-a web-based toolset for functional profiling of gene lists from large-scale experiments. Nucleic Acids Res..

