# Supplementary material for: A point mutation in HIV-1 integrase redirects proviral integration into centromeric repeats
Source: Nat Commun. 2022 Mar 18;13:1474. doi: 10.1038/s41467-022-29097-8 (PMC8933506; doi:10.1038/s41467-022-29097-8)
Supplement: Supplementary file 1 — Supplementary Information [file 41467_2022_29097_MOESM1_ESM.pdf]

Supplementary information

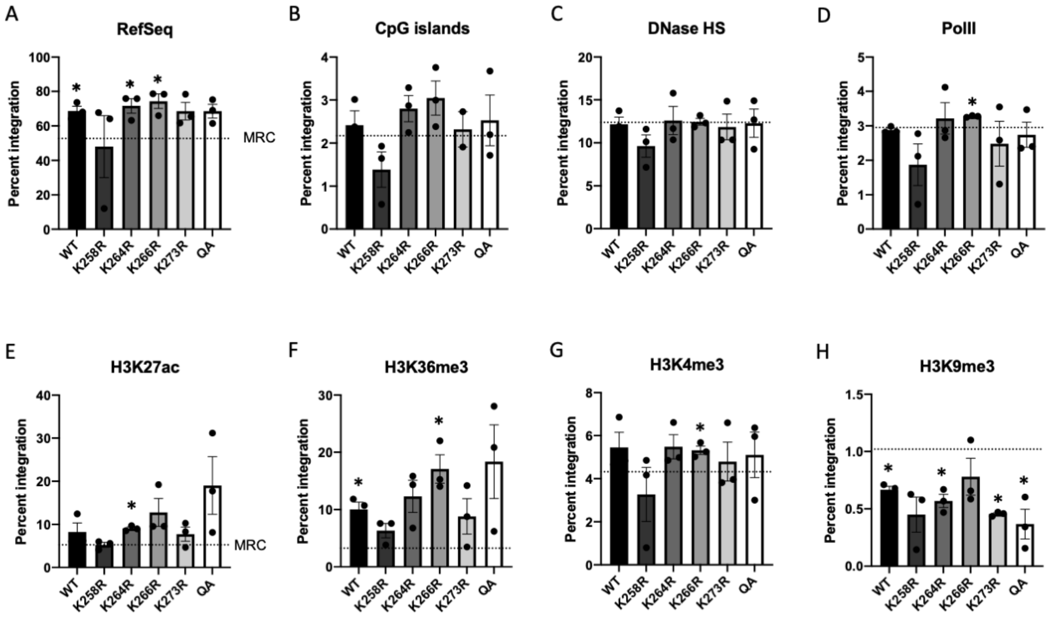

Figure S1: Integration frequency of WT and acetylation mutant IN proteins with respect to common genomic features. Frequency of integrations falling within (A) RefSeq genes, or within 1 kb of (B) CpG islands, (C) DNase hypersensitivity sites, (D) RNA polymerase II binding sites and various pre-infection histone modification sites in HeLa cells (E-H) was calculated using BedTools. Frequency of integrations in a matched random control (MRC) data set is shown as dashed line. Data is shown as the average of three independent replicates +/- SEs. Statistical significance of integration frequency relative to MRC was gauged by a one-sample, two tailed t-test (*p<0.05, all p-values shown in Table S3). Source data are provided as a Source Data file.

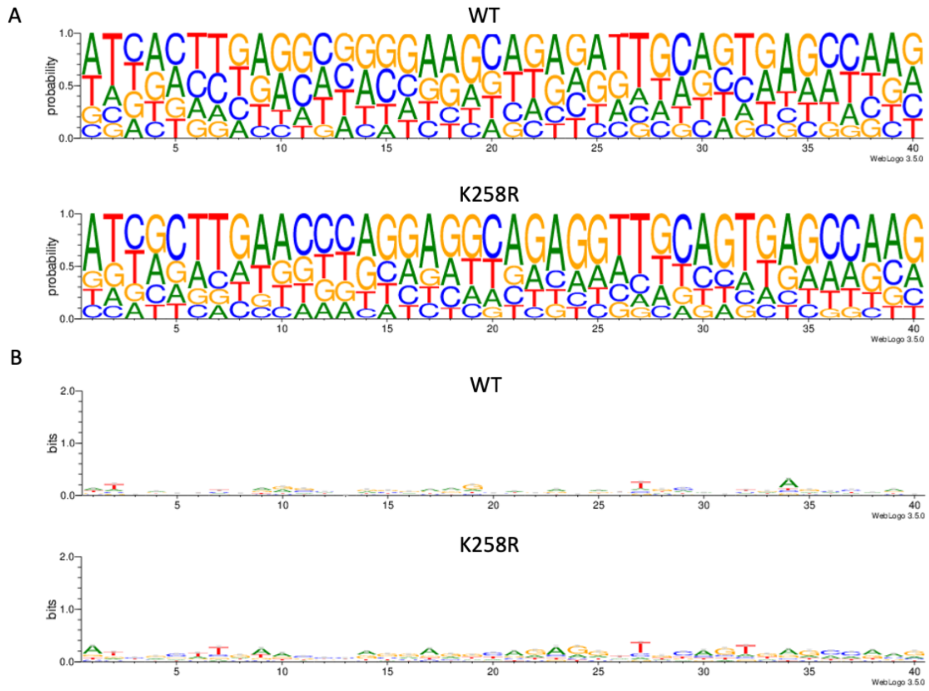

Figure S2: Consensus sequence around the site of integration for viruses carrying WT or K258R mutant. Consensus sequence around the site of integration (+/-20 bp) was generated by MEME for integrations generated by WT or K258R IN depicted as (A) probability of nucleotide at each position or (B) as a function of entropy. Cumulative data from three independent biological replicates was used for analysis.

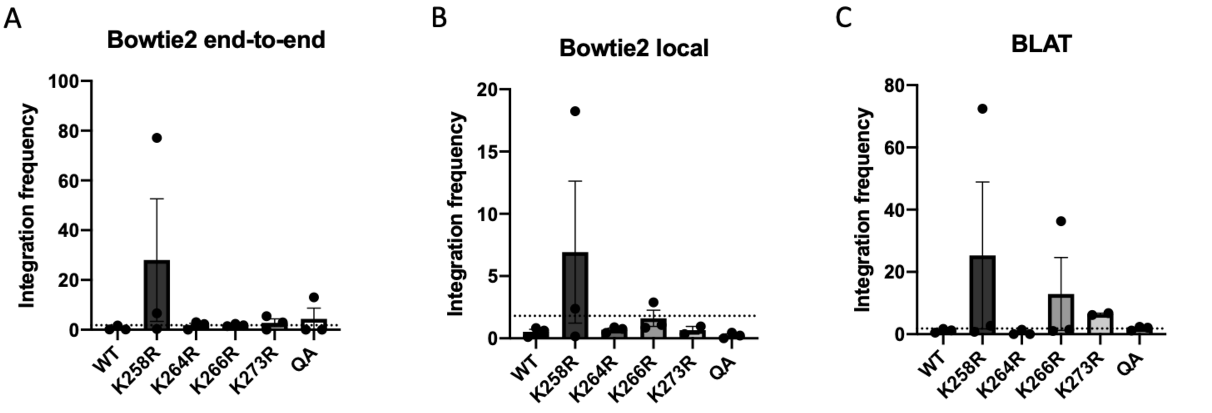

	Total mapped integrations	Integrations mapped to centromeres
WT	17636	146
K258R	18850	1576
K264R	16890	226
K266R	11947	206
K273R	8168	123
QA	16565	154
MRC	10000	184

	Total mapped integrations	Integrations mapped to centromeres
WT	45873	204
K258R	51282	993
K264R	45051	309
K266R	50084	530
K273R	16916	59
QA	42064	93
MRC	50000	968

	Total mapped integrations	Integrations mapped to centromeres
WT	30637	277
K258R	33929	2586
K264R	19879	176
K266R	32248	492
K273R	2130	143
QA	25138	425
MRC	30000	617

Figure S3: Integration frequency into centromeres using different mapping algorithms. NGS data from three independent biological replicates was mapped to the GRCh38 human genome assembly using (A) Bowtie2 end-to-end alignment (shown in main text), (B) Bowtie2 sensitive local alignment or (C) BLAT alignment algorithms. Bar graphs show the integration frequency into centromeres as determined by each algorithm. Graphed data is presented as an average of three independent biological replicates +/- SEs. Absolute number of detected unique integrations for all libraries summed is shown below each graph. Source data are provided as a Source Data file.

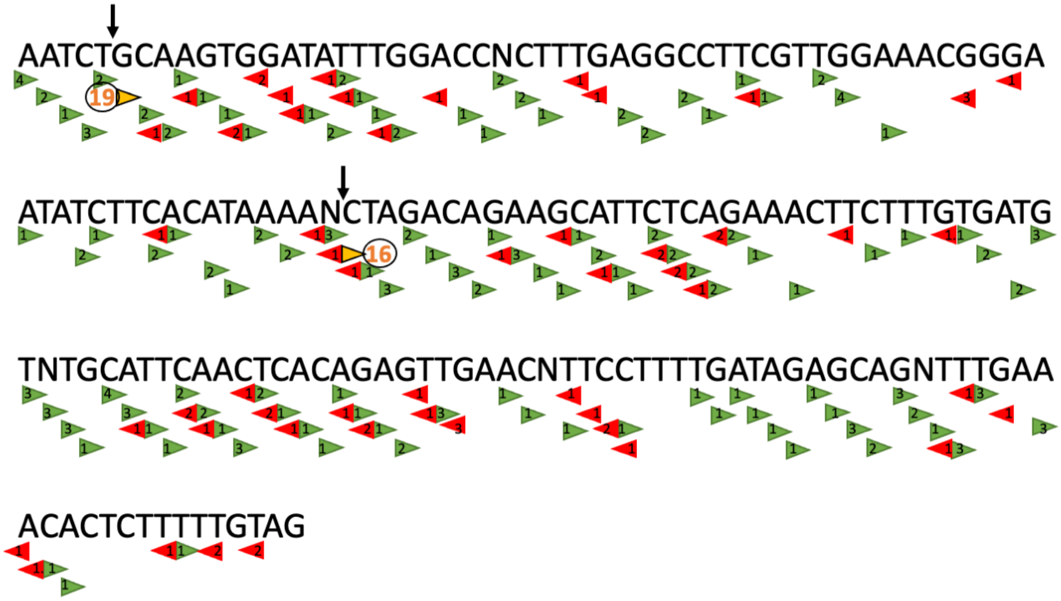

Figure S4: Schematic depicting integration events into alphoid repeat monomer consensus sequence. Shown are the exact sites of integration for each unique integration event that mapped to the alphoid repeat sequence from three independent experiments. The number of integrations at each location as well as their orientation is noted. Arrows point to hot-spot locations of integration.

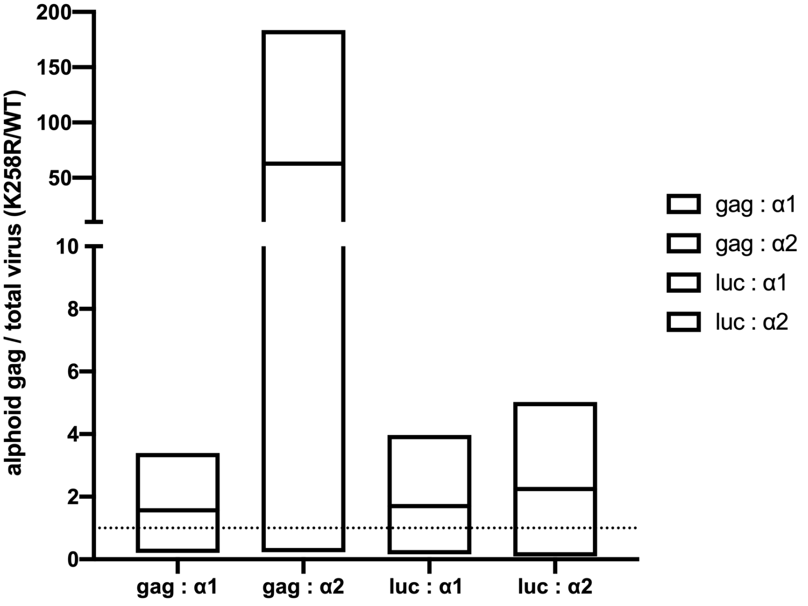

Fig S5: K258R mutant virus integrates near alphoid repeats more often than WT virus in Jurkat cells. Integration into centromeric alphoid repeat DNA was quantified using a alphoid-virus nested PCR approach as described in Fig. 5. First round PCR was performed with primers in the alphoid repeat (α1, α2) and primers in either the 5' end of *gag* or the 3' end of the luciferase reporter gene. Shown are the results of a second round nested quantitative PCR using LTR specific primers normalized to total virus levels. Data from three independent replicates is shown relative to WT as box plots to show the minimum, maximum and mean values. Source data are provided as a Source Data file.

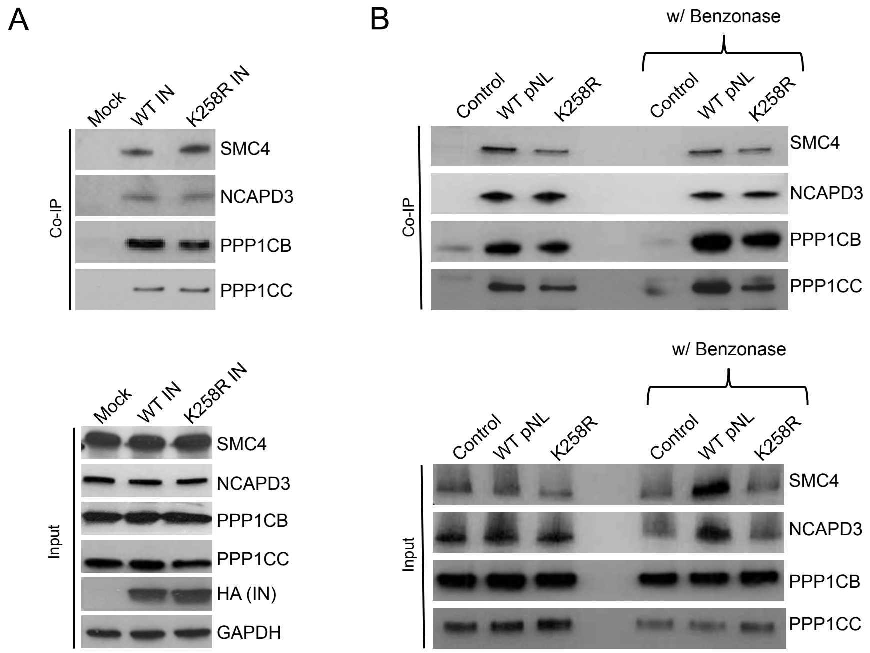

Figure S6: Validation of candidate host factor binding to WT and K258R mutant IN proteins. (A) Upper panel: HA-tagged IN proteins were immunoprecipitated from HEK293T cells after transfection with plasmids expressing either WT or K258R mutant IN, or mock transfected. Immunoprecipitated proteins were analyzed by Western blots probed with antisera specific for the indicated candidate host factors. Lower panel: Proteins present in the lysates input to the immunoprecipitation probed with the indicated antisera. (B) Repeat of coimmunoprecipitation as in panel A, performed with addition of benzonase to the lysate to digest RNA and DNA, as indicated. Three independent replicates were performed, representative blots shown. See Source Data for unedited blots.

<u>Table S1</u>: Statistical analysis of integration preferences of K258R mutant IN protein as compared to WT (paired t-test or Fisher's exact test, two-tailed, significance level of p = 0.05).

	**Paired t-test (N=3)**	**Fisher's exact test**
RefSeq genes	0.3247	<0.0001
TSS	0.1691	<0.0001
CpG islands	0.1547	<0.0001
RNA Pol II	0.2656	0.0051
DNase HS	0.3578	0.0.006
H3K27ac	0.3797	<0.0001
H3K36me3	0.2531	<0.0001
H3K4me3	0.3805	0.0025
H3K9me3	0.2278	0.1847
Centromeric	0.3860	<0.0001

Table S2: Host proteins immunoprecipitated with WT or K258R mutant IN protein.

Sample(s)	Host protein names
WT/K258R common binding partners	PRKDC, MDN1, MYBBP1A, NUP205, CAND2, GCN1, NUP188, CKMT1A, IMMT, HEATR1, IPO4, UBE3C, AIFM1, FANCI, ABCD3, ATP2A2, ABCE1, LTN1, SUCLA2, COQ8B, ATAD3C, DDX20, AFG3L2, ATAD3A, ATAD3B, RCN2, SGPL1, TYK2, SLC16A1, MCM7, TIMM50, ARF4, RRP12, PPP1CB, SLC25A10
WT specific binding partners	TEX10, GEMIN4, UNC45A, YME1L1, ARF5, NOP56, EIF2S2, RPL27
K258R specific binding partners	NUP93, JAK1, NCAPD3, GLUD1, CHCHD3, SPATA5, CAND1, TMEM209, PLEKHG4, RPP30, HACD3, ILVBL, SMC4, RPSKA4, CAD, ALDH1B1, RPN1, PPP1CC, ATP1A1, HERC5, RTCB

<u>Table S3</u>: Gene ontology analysis of integrase interacting host factors. GO analysis of either all binding partners, or only specific binding partners was performed for WT and K258R IN proteins.

GO category	# of proteins	P-value	Proteins
WT (all binding partners)			
Nucleotide binding	19	5.4E-5	PRKDC, MDN1, CKMT1A, AIFM1, ABCD3, ATP2A2, ABCE1, SUCLA2, COQ8B, ATAD3C, DDX20, AFG3L2, ATAD3A, ATAD3B, TYK2, MCM7, ARF4, YME1L1, ARF5
WT (specific binding partners)			
rRNA processing	4	1.7E-3	TEX10, GEMIN4, NOP56, RPL27
K258R (all binding partners)			
Nucleotide binding	26	5.2E-8	JAK1, GLUD1, SPATA5, SMC4, RPS6KA4, CAD, ALDH1B1, ATP1A1, RTCB, PRKDC, MDN1, CKMT1A, AIFM1, ABCD3, ATP2A2, ABCE1, SUCLA2, COQ8B, ATAD3C, DDX20, AFG3L2, ATAD3A, ATAD3B, TYK2, MCM7, ARF4
Antiviral mechanism by IFN-stimulated genes	6	2.1E-4	NUP93, JAK1, HERC5, NUP205, ABCE1, NUP188
tRNA processing in the nucleus	5	8.3E-4	NUP93, RPP30, RTCB, NUP205, NUP188
PTW/PP1 complex	2	4.9E-2	PPP1CB, PPP1CC
K258R (specific binding partners)			
tRNA processing	3	2.3E-2	RPP30, RTCB, NUP93
ISG15 antiviral mechanism	3	4.7E-2	JAK1, HERC5, NUP93
Meiotic chromosome condensation / condensin complex	2	5.2E-3	NCAPD3, SMC4

Table S4: Primer sequences used for quantitative PCR analysis of viral DNA intermediates and transcripts.

Target	Primer sequence (5’-3’)
Late RT	TGTGTGCCCGTCTGTTGTGT
	GAGTCCTGCGTCGAGAGATC
Luciferase	CGTCTTTCCGTGCTCCAAAAC
	CAAAGGATATCAGGTGGCCC
2LTR circles	AACTAGGGAACCCACTGCTTAAG
	TCCACAGATCAAGGATATCTTGTC
Alu-gag nest 1	GCCTCCCAAAGTGCTGGGATTACAG
	GCTCTCGCACCCATCTCTCTCC
Alu-gag nest 2	GCCTCAATAAAGCTTGCCTTGA
	TCCACACTGACTAAAAGGGTCTGA
Tat mRNA	GTTTGTTTCATGACAAAAGCCTTA
	CTATTCCTTCGGGCCTGTC
Chr1	GTTCCCTTAGACAGAGCAGATTT
	CAACGCAGTTTGTGGGAATG
Chr2	TCGTTGGAAACGGGATTGT
	CTGCTCTATGAAAGGGACTGTT
Chr4	CTGTAGTATCTGGAAGTGGACATT
	GGTTCAACTGTGTTCGTTTAGG
Chr14	GATTTCGTTGGAAACGGGATTAC
	AGAAAGATCCACGCCTGTTA
Alphoid-1	GCAAGGGGATATGTGGACC
Alphoid-2	ACCACCGTAGGCCTGAAAGCAGTC
5’-gag	GCTCTCGCACCCATCTCTCTCC
3’-luc	AGGCCAAGAAGGGCGGAAAG

Table S5: Adaptor and primer sequences used for construction of integration site mapping NGS libraries.

Primer name	Primer sequence
Adaptor short arm	P-GATCGGAAGAGCAAAAAAAAAAAAAAAA
Adaptor long arm	CAAGCAGAAGACGGCATACGAGATnnnnnnGTGACTGGAGTTCAGACGTGTGCTCTTCCGATC*T
PCR-1-F	TGTGACTCTGGTAACTAGAGATCCCTC
PCR-1-R	CAAGCAGAAGACGGCATACGAGAT
PCR-2-F	AATGATACGGCGACCACCGAGATCTACACTCTTTCCCTACACGACGCTCTTCCGATCTGAGATCCCTCAGACCCTTTTAGTCAG
PCR-2-R	CAAGCAGAAGACGGCATACGAGATnnnnnn

nnnnnn denotes a 6-bp unique barcode, P denotes phosphorylation and * denotes a phosphorothioate bond

Table S6: Statistical analysis of integration preferences of WT or mutant IN proteins as compared to a matched random control (one sample two-tailed t-test, significance level of p = 0.05).

	**WT**	**K258R**	**K264R**	**K266R**	**K273R**	**QA**
RefSeq genes	0.0285	0.8183	0.0463	0.0356	0.0885	0.058
TSS	0.4319	0.1111	0.0197	0.0489	0.2596	0.0849
CpG islands	0.5413	0.1959	0.1735	0.1587	0.7776	0.6058
RNA Pol II	0.3183	0.2166	0.6193	0.0018	0.5446	0.6232
DNase HS	0.8181	0.1693	0.9057	0.8804	0.7553	0.6058
H3K27ac	0.2927	0.9830	0.0079	0.1451	0.2675	0.1759
H3K36me3	0.0336	0.1331	0.0837	0.0304	0.2136	0.1432
H3K4me3	0.2517	0.4915	0.1764	0.0356	0.6527	0.535
H3K9me3	0.0073	0.0653	0.016	0.2742	0.0003	0.0372
Centromeric	0.1580	0.3991	0.9515	0.8294	0.598	0.6204
