# Supplementary material for: A point mutation in HIV-1 integrase redirects proviral integration into centromeric repeats
Source: Nat Commun. 2022 Mar 18;13:1474. doi: 10.1038/s41467-022-29097-8 (PMC8933506; doi:10.1038/s41467-022-29097-8)
Supplement: Supplementary file 2 — Reporting Summary [file 41467_2022_29097_MOESM2_ESM.pdf]

nature research

Corresponding author(s): Stephen P. Goff

Last updated by author(s): Dec 21, 2021

Reporting Summary

Nature Research wishes to improve the reproducibility of the work that we publish. This form provides structure for consistency and transparency in reporting. For further information on Nature Research policies, see our Editorial Policies and the Editorial Policy Checklist.

Statistics

For all statistical analyses, confirm that the following items are present in the figure legend, table legend, main text, or Methods section.

n/a	Confirmed	
☐	☒	The exact sample size (*n*) for each experimental group/condition, given as a discrete number and unit of measurement
☐	☒	A statement on whether measurements were taken from distinct samples or whether the same sample was measured repeatedly
☐	☒	The statistical test(s) used AND whether they are one- or two-sided<br>*Only common tests should be described solely by name; describe more complex techniques in the Methods section.*
☒	☐	A description of all covariates tested
☐	☒	A description of any assumptions or corrections, such as tests of normality and adjustment for multiple comparisons
☐	☒	A full description of the statistical parameters including central tendency (e.g. means) or other basic estimates (e.g. regression coefficient) AND variation (e.g. standard deviation) or associated estimates of uncertainty (e.g. confidence intervals)
☒	☐	For null hypothesis testing, the test statistic (e.g. *F*, *t*, *r*) with confidence intervals, effect sizes, degrees of freedom and *P* value noted<br>*Give P values as exact values whenever suitable.*
☒	☐	For Bayesian analysis, information on the choice of priors and Markov chain Monte Carlo settings
☒	☐	For hierarchical and complex designs, identification of the appropriate level for tests and full reporting of outcomes
☒	☐	Estimates of effect sizes (e.g. Cohen's *d*, Pearson's *r*), indicating how they were calculated

Our web collection on statistics for biologists contains articles on many of the points above.

Software and code

Policy information about availability of computer code

Data collection	Next generation high throughput sequencing was performed using Illumina MiSeq.
Data analysis	Bowtie2 (Galaxy Version 2.3.4.3)<br>BLAT (v407)<br>Clustal Omega Multiple Sequence Alignment<br>g:Profiler (version e101_eg48_p14_baf17f0)<br>BedTools (v 2.29.2)<br>GraphPad Prism (Version 9.0)<br>Common integration site analysis - custom perl script adapted from: Malhotra, S. et al. Selection for avian leukosis virus integration sites determines the clonal progression of B-cell lymphomas. PLoS Pathog 13, (2017).

For manuscripts utilizing custom algorithms or software that are central to the research but not yet described in published literature, software must be made available to editors and reviewers. We strongly encourage code deposition in a community repository (e.g. GitHub). See the Nature Research guidelines for submitting code & software for further information.

Data

Policy information about availability of data

All manuscripts must include a data availability statement. This statement should provide the following information, where applicable:

- Accession codes, unique identifiers, or web links for publicly available datasets
- A list of figures that have associated raw data
- A description of any restrictions on data availability

Data regarding sequence annotation of the reference genome was obtained from UCSC Genome Browser. Publicly available ChIP-seq datasets from ENCODE were

used to obtain locations of protein binding sites in HeLa cells (Accession numbers listed in methods section for each relevant dataset). NGS data produced in this study will be submitted to NCBI SRA before publication. Any additional data is available upon contact.

Field-specific reporting

Please select the one below that is the best fit for your research. If you are not sure, read the appropriate sections before making your selection.

- [x] Life sciences
- [ ] Behavioural & social sciences
- [ ] Ecological, evolutionary & environmental sciences

For a reference copy of the document with all sections, see nature.com/documents/nr-reporting-summary-flat.pdf

Life sciences study design

All studies must disclose on these points even when the disclosure is negative.

Sample size	Sample size was based on traditional experimental design approach in molecular and cellular biology. In general, qPCR, luciferase and NGS experiments were repeated a minimum of 3 independent times.
Data exclusions	No data was excluded from this study.
Replication	Each experiment was replicated a minimum of three times. Each biological replicate was completely independent (from new cell and virus preparations). Within each biological replicate, there were also technical replicates on the same samples. Due to the variability in the extent of the phenotype, large scale next-generation sequencing experiments were also performed three independent times and sequenced in independent MiSeq runs.
Randomization	The experiments did not require randomization. Samples were handled identically in all experiments.
Blinding	The investigators were not blinded to group allocation during data collection or subsequent analysis. This approach is considered standard for biochemical experiments performed here.

Reporting for specific materials, systems and methods

We require information from authors about some types of materials, experimental systems and methods used in many studies. Here, indicate whether each material, system or method listed is relevant to your study. If you are not sure if a list item applies to your research, read the appropriate section before selecting a response.

Materials & experimental systems

n/a	Involved in the study	
☐	☒	Antibodies
☐	☒	Eukaryotic cell lines
☒	☐	Palaeontology and archaeology
☒	☐	Animals and other organisms
☒	☐	Human research participants
☒	☐	Clinical data
☒	☐	Dual use research of concern

Methods

n/a	Involved in the study	
☒	☐	ChIP-seq
☒	☐	Flow cytometry
☒	☐	MRI-based neuroimaging

Antibodies

Antibodies used	anti-HA (abcam ab9110), anti-IN (abcam ab 66645), Pierce anti-HA magnetic beads (ThermoFisher 88837).
Validation	Anti-HA and anti-IN antibodies were used to ensure equal expression of WT and mutant IN proteins from expression vectors used for IP experiment. These were validated using a Western blot with negative control (no HA-protein expression). Also validated by size of protein detected on blot and by comparability between the detection of the same tagged IN protein by both antibodies on the same blot.<br><br>Pierce magnetic anti-HA beads were used for actual IP. Negative controls used in IP - non-transfected cells and cells transfected with empty vector.

Eukaryotic cell lines

Policy information about cell lines

Cell line source(s)	HeLa (CCL-2), HEK293T (CRL-11268) cell lines were obtained from ATCC.
Authentication	Authenticated by ATCC; cell morphology confirmation by microscope.
Mycoplasma contamination	Cell lines were not tested for Mycoplasma contamination
Commonly misidentified lines (See ICLAC register)	No commonly misidentified cell lines were used.
